# Compact Waveguide Antenna Design for 77 GHz High-Resolution Radar

**DOI:** 10.3390/s25113262

**Published:** 2025-05-22

**Authors:** Chin-Hsien Wu, Tsun-Che Huang, Malcolm Ng Mou Kehn

**Affiliations:** 1 Wistron NeWeb Corporation, Hsinchu 300092, Taiwan; tc.huang@wnc.com.tw; 2 Institute of Communications Engineering, National Yang Ming Chiao Tung University, Hsinchu 300093, Taiwan; malcolm.ng@ieee.org

**Keywords:** millimeter-wave, narrow-wall slots, open-ended waveguide, slotted waveguide antenna

## Abstract

Millimeter-wave antennas have become more important recently due to the diversity of applications in 5G and upcoming 6G technologies, of which automotive systems constitute a significant part. Two crucial indices, detection range and angular resolution, are used to distinguish the performance of the automotive antenna. Strong gains and narrow beamwidths of highly directive radiation beams afford longer detection range and finer spatial selectivity. Although conventionally used, patch antennas suffer from intrinsic path losses that are much higher when compared to the waveguide antenna. Designed at 77 GHz, presented in this article is an 8-element slot array on the narrow side wall of a rectangular waveguide, thus being readily extendable to planar arrays by adding others alongside while maintaining the element spacing requirement for grating lobe avoidance. Comprising tilted Z-shaped slots for higher gain while keeping constrained within the narrow wall, adjacent ones separated by half the guided wavelength are inclined with reversed tilt angles for cross-polar cancelation. An open-ended external waveguide is placed over each slot for polarization purification. Equivalent circuit models of slotted waveguides aid the design. An approach for sidelobe suppression using the Chebyshev distribution is adopted. Four types of arrays are proposed, all of which show potential for different demands and applications in automotive radar. Prototypes based on designs by simulations were fabricated and measured.

## 1. Introduction

As modern society becomes prosperous with the rapid development of the economy, the associated advancements of the current 5G towards future 6G technologies are inevitable. There are increasing demands for enhanced data speeds, accuracy, bandwidths, spectral and energy efficiencies, throughputs, capacities, as well as signal quality and reliability [1,2]. Lower latencies, wider coverage through massive connectivities, improved security, as well as reduced interferences, multipath fading, and path losses, are also sought. Applications include communications, navigation, radar, tracking, imaging, detection, positioning, localization, and sensing. One other important sector not to be omitted is of course the automotive industry as commuting has become an indispensable part in our daily lives [3]. However, the rising amounts of vehicles on the roads lead to traffic jams and higher rates of accidents, thus raising the awareness of driving management and security. As prevention is better than cure, the technology of automotive radar system provides methods for reducing the risk of occurrence of these issues.

The frequency bands of automotive radars can be divided into the 24 GHz for short-range (SRR) radar and mid-range radar (MRR), and the 77 GHz bands for long-range radar (LRR) [4]. There are advantages of the 77 GHz band over the one centered about 24 GHz. For instance, the former has a larger available bandwidth of 4 GHz while the 24 GHz band has only 250 MHz bandwidth [5]. Because the resolution and accuracy are inversely proportional to the wavelength, as the latter becomes smaller from 24 GHz to 77 GHz band, the resolution and accuracy improve by a factor of three. Another advantage is size reduction. As the wavelength at 77 GHz is about a third that of 24 GHz, an antenna operating at 77 GHz is about one-ninth of the size of one designed for 24 GHz. Devices operating in the 77 GHz band also have higher levels of transmitted powers [6].

The slotted waveguide antenna is a radiating element that has been widely used in various applications such as radar and communication systems [7]. When it comes to radar systems, the commonly used devices include designs based on waveguides, groove gap waveguides [8], or microstrip antennas. Waveguides possess some outstanding characteristics such as low loss and high efficiency as compared to the other aforementioned prototypes [8]. Unlike microstrip line excitation that suffers from substrate dielectric and conducting patch losses, the waveguide is a comparatively much less dissipative structure as shown in Table 1 [8].

For a radar antenna with gain G relative to an isotropic radiator, assuming the distances from the target to the transmitter and receiver are equal, the detectable range *R_max_* is proportional to the square root of the antenna gain *G*: [9]

(1) $$R_{max}=\sqrt[{4}]{\frac{P_{s}G^{2}\lambda^{2}\sigma }{{\left(4\pi \right)}^{3}P_{e\_min}}}$$

where *P_s_* is the transmitted power, *λ* is the wavelength, *σ* is the radar cross section, and *P_e_min_* is the minimum received power. Therefore, for a 10 cm structure, the loss of the rectangular waveguide is 6 dB smaller than the microstrip line, which indicates the detection range of the rectangular waveguide is two times further than the microstrip line. This idea brought out our objectives of this research, the main one of which is to replace the microstrip line trace with the rectangular waveguide, enhancing the antenna gain and increasing the detection range.

**Figure 1:**
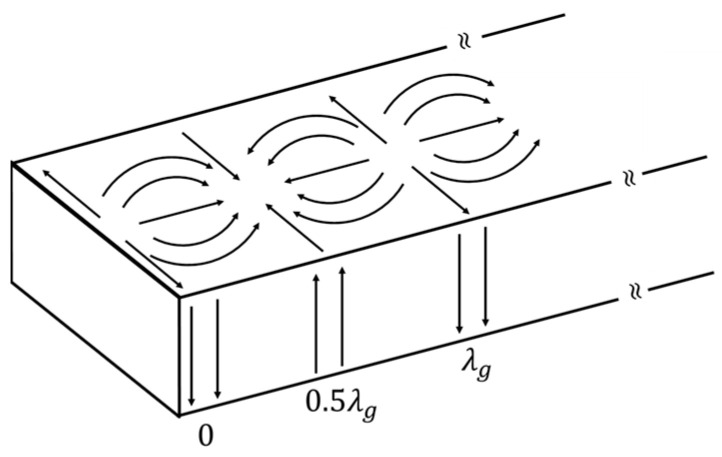
Surface current on guide walls for *TE*_10_ mode in a rectangular waveguide. Arrows denote the direction of surface current.

Three types of slots implemented on waveguides would be discussed individually. For longitudinal slots implemented on the broad walls of rectangular waveguides [10] so as to perpendicularly cut the currents along the waveguide width that connect with those along the waveguide height on the narrow walls, the distance between adjacent slots along the axis direction is 0.5*λ_g_*, where *λ_g_* is the guided wavelength in the waveguide. The design is such that this 0.5*λ_g_* slot spacing is still less than the free space wavelength *λ*_0,_ so as to avoid grating lobes for just broadside radiation despite *λ_g_* being larger than *λ*_0_, i.e., *λ*_0_ *< λ_g_ <* 2*λ*_0_. However, as seen from the electric surface currents on the conducting walls of a rectangular waveguide in Figure 1, the direction reverses every 0.5*λ_g_*, which would lead to out-of-phase radiators if the slots are all placed on one side with respect to the center axis. Therefore, by assigning an opposite offset to the adjacent slots with reference to the central axis line to attain a further *π* phase variation along the waveguide width, the in-phase condition for broadside radiation would be achieved.

As for longitudinal slots implemented on the narrow wall of the waveguide [11], the direction of the electric current reverses every 0.5*λ_g_* and cycles back to the same direction every *λ_g_*. Therefore, for in-phase slot radiators, the spacing of longitudinal slots on the narrow wall of waveguides (*λ_g_*) is two times larger than that on the broad wall (*λ_g_*), which violates the spacing for avoidance of grating lobes even for just broadside radiation. However, there had been research which demonstrated how to reduce the spacing of the narrow-wall slot to 0.5*λ_g_*. Among these studies, one method is by placing several separate waveguides side-by-side to form a planar waveguide array, with an equivalent spacing of 0.5*λ_g_* [12,13]. Another technique is by etching a tilted parasitic dipole on the dielectric substrate while the un-tilted slot is on the other side of the substrate [14]. Because the tilted dipole rotates the *E*-field in the waveguide, it excites the slot and generates the *E*-field successfully. By rotating the tilted dipole in a clockwise and counterclockwise order, the vertical *E*-field is canceled and the horizontal one is summed up. This concept enables the *E*-field to fulfill the in-phase condition. The third approach is by embedding slots on the narrow wall of the waveguide without a dipole [15]. The difference in this method from the second one is that the tilted element is a slot itself rather than a dipole. The principle of the third method is similar to the second one, which also generates horizontally and vertically oriented *E*-fields. As a result, by means of oblique slots, 0.5*λ_g_* spacings can be achieved as well. The third method is used in this research.

After discussing the electric surface current distributions on the walls of rectangular waveguides and the periodicities of slot arrangements, another aspect would be the slot design, which is related to the conductance value. This theory can be traced back to the 1970s [16]. An inclined slot is too long to implement on the narrow side of a rectangular waveguide; therefore, an alternative solution is to bend the slot into deformed versions such as the H-shaped, Z-shaped, and I-shaped slots, and confining the slot to the narrow wall. The details of the conductance calculations are discussed in the next section. In [17], while not affecting the performance of slot array, the Z-shaped slot can also provide better structural strength because the distance from the slot edges to the corner of the waveguide is further. As another application of the Z-shaped slot, it can also be used as a transition or coupling element between different layers [18].

There are a variety of slot shapes for slot array antenna, as shown in Table 2. The slots in [19,20,21,22] are embedded on the broad wall of a waveguide. Due to either the circular shapes or the sharp angles of the slot elements, difficulties with fabrication are incurred. Similar to this work, ref. [14] is also a narrow wall slot antenna with spacing of $0.5\lambda_{g}$. The I-shape slot can be comparable to our Z-shaped slot. While our Z-shaped slot is fed by a low-loss waveguide, the I-shaped slots are excited by dipoles, which are mounted on a PCB layer. This layer results in additional losses at high frequencies and increased fabrication complexities constituting the main differences.

**Figure 2:**
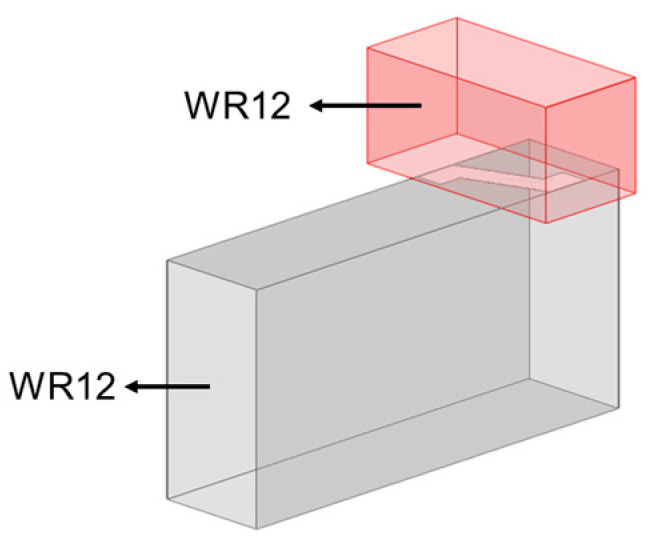
Z-shaped slot in narrow-wall waveguide with an open-ended waveguide.

In this paper, the vital feature of this radiating design would be implementing, on the narrow side wall of a rectangular waveguide, an array of inclined Z-shaped slots with alternately reversed tilt angles for cancelation of X-pol. radiation components, and adding an open-ended waveguide above each slot for polarization purification, as shown in Figure 2. Array slotting of the narrow side wall allows for the compact placement of other narrow-wall arrays side-by-side to realize 2D planar arrays that more readily fulfill the periodicity requirements for avoidance of grating lobes. Moreover, an array with element spacing of 0.5*λ_g_* brings up a potential competence for a compact multi-array structure and less routing complexity.

In Section 2.1, the reason for the use of a Z-shaped slot element instead of a straight slot will be described. Discussions about the placement of an open-ended waveguide over each slot to purify the polarization are given in Section 2.2. Presented in Section 2.3 is the concept of using the Chebyshev distribution to suppress the sidelobes. In Section 3, the prototype of our proposed structure shall be described and the results of its simulations will be presented. In Section 4, experiments conducted on a fabricated prototype are reported, the measurement results of which show good consistency with the simulation results. Finally, concluding remarks and a summary of the work are given in Section 5.

## 2. Theory

### 2.1. Z-Shaped Slot Coupling

**Figure 3:**
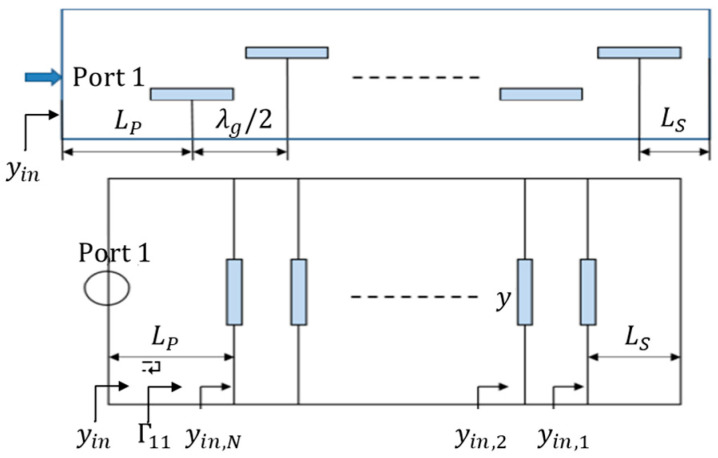
Slotted waveguide and its equivalent circuit [24]. Single head arrows denote the normalized input admittance.

Combining the concept of the ABCD-matrix [23] and equivalent circuit models of slotted waveguides [24], the admittance of the slot can be calculated, the real and imaginary parts of which being the conductance (*G*) and susceptance (*B*), respectively. The slotted waveguide and its equivalent circuit model are shown in Figure 3 [24], where *L_S_* is the distance from the first (rightmost) slot to the short-circuit matching stub, and *L_P_* is the distance from the last (leftmost) slot to the input port.

According to the transmission line theory, the normalized input admittance is as follows:

(2) $$y_{in}\left(L\right)=[y_{l}+j\tan\left(\beta L\right)]/[1+jy_{l}\tan\left(\beta L\right)]$$

where *L* is the distance of the input from the normalized load admittance *y_l_*, and *β* is the propagation constant. When having a short-circuit load condition (*y_l_ = ∞*), and in the context of Figure 3, where *L* is set to *L_S_*, (2) can be written as follows:

(3) $$y_{in,sc}\left(L_{s}\right)=1/[j\tan\left(\beta L_{s}\right)]$$

Assuming all slots are the same, the normalized slot admittance of each denoted as *y*, the normalized input admittance *y_in,n_* at any *n-th* slot can be expressed as follows:

(4) $$y_{in,n}=[y_{in,n-1}+j\tan\left(\beta \lambda_{g}/2\right)]/[1+jy_{in,n-1}\tan\left(\beta \lambda_{g}/2\right)]+y;\;\;2\;\leq n\leq \;N$$

(5) $$y_{in,1}=y_{in,sc}\left(L_{s}\right)+y$$

The normalized input admittance at the input port is then:

(6) $$y_{in}=\left[y_{in,n}+j\tan\left(\beta L_{p}\right)\right]/[1+jy_{in,n}\tan\left(\beta L_{p}\right)]$$

When *n* = 1, the normalized input admittance can be expressed by the reflection coefficient Γ_11_ = *S*_11_ [25]:

(7) $$y_{in}=\left[1-\Gamma_{11}\exp\;(j2\beta L_{p})\right]/\left[1+\Gamma_{11}\exp\;(j2\beta L_{p})\right]$$

Therefore, the normalized slot admittance can be determined by (3), (5), and (7):

(8) $$y=\left[1-\Gamma_{11}\exp\;(j2\beta L_{p})\right]/[1+\Gamma_{11}\exp\;(j2\beta L_{p})]+j\cot\left(\beta L_{s}\right)$$

where, *β =* 2*π/λ_g_*, and *λ_g_* is the guide wavelength in the waveguide. When *L_P_ =* 2*λ_g_*, *L_S_ = λ_g_*/4:

(9) $$y=\left[1-S_{11}\right]/[1+S_{11}]$$

Thus, the normalized slot admittance can be calculated in HFSS by means of the S-parameter under these conditions.

**Figure 4:**
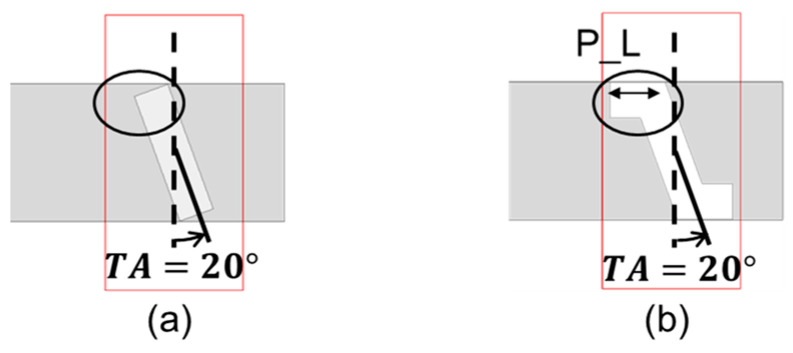
Increasing slot length from straight slot to Z-shaped slot. (**a**) Straight slot; (**b**) Z-shaped slot.

The waveguide of the proposed antenna is the WR12 standard waveguide, with cross-sectional dimensions 3.10 × 1.55 (mm^2^). Thus, for the narrow-wall slotted waveguide, the slot length is confined. However, when the slot length is not long enough, the slot is unable to radiate well. The excitation mechanism of the proposed antenna is by rotating the slots at different tilt angles *TA*(°) made with the direction along the dimension of the narrow wall, as depicted in Figure 4a. As the tilt angle increases, the slot length can become longer within the same waveguide. However, as our simulation results have shown, the slot is still unable to radiate well even with larger tilt angles. In order to solve this problem, an intuitive remedy would be to increase the slot length. This is herein achieved by deforming the slot into the form of a Z-shaped slot, thereby extending the slot along the axis direction, as shown in Figure 4b. The parameter P_L denotes the length of each parallel part of the Z-shaped slot.

**Figure 5:**
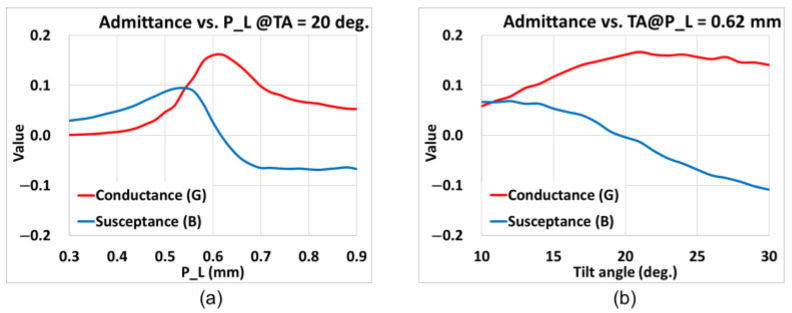
Graphical relation between the normalized slot conductance (G) and susceptance (B) and *TA* via *P_L* at 77 GHz at (**a**) *TA* = 20° and (**b**) *P_L* = 0.62 mm.

The normalized slot admittance is calculated at 77 GHz. By progressively changing *P_L* and thus the total slot length under a fixed *TA*, the characteristic relation between the normalized slot admittance and the slot length can be established and plotted, as shown in Figure 5a, in which the red and blue lines respectively represent the conductance and susceptance. Similarly, as shown in Figure 5b, the corresponding characteristic relational plot can be acquired by modifying the tilt angle *TA* with a fixed slot length *P_L* = 0.62 mm. Based on these results, the Z-shaped slot effectively lengthens the slot and allows the slot to radiate.

### 2.2. Polarization Purification by Open-Ended Waveguides

**Figure 6:**
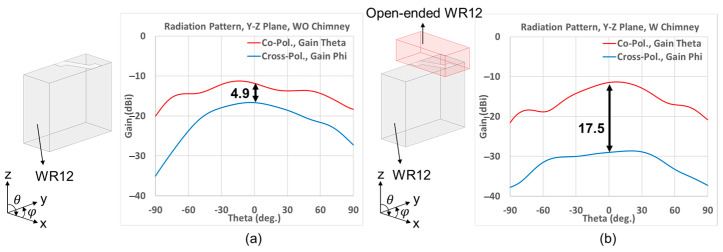
Graphs of gain versus theta at 77 GHz, with the theta and phi gain components being the co-pol. and X-pol., respectively, as indicated, (**a**) poor X-pol. isolation with slot directly etched on waveguide, and (**b**) X-pol. radiation mitigated with an open-ended waveguide covering the slot.

As the fields pass through an embedded Z-shaped slot on the waveguide wall, the resultant radiation pattern at 77 GHz of this radiating element has a poor X-pol. of about 4.9 dB, as shown in Figure 6a. The obvious reason for this poor X-pol. isolation lies with the two orthogonal components (vertical and horizontal), which the *E*-field over the tilted portion of the Z-slot is decomposed into.

In order to enhance the X-pol. isolation, an open-ended waveguide is placed over the slot with its width in the *x*-direction, height in the *y*-direction (width is larger than height), length in the *z*-direction of wave propagation, and with its width oriented along the height dimension of the waveguide with the narrow-wall slots, as shown by the inset diagram of Figure 6b. By means of this open-ended waveguide, only one of the two orthogonal components into which the *E*-field within the tilted part of the Z-slot is decomposed gets to be transported by the sole propagating *TE*_10_ mode up the output waveguide for eventual aperture radiation, but not the other polarization associated with the next higher order *TE*_01_ mode that is designed to be evanescent. As a result, modes in two directions can be extracted for operations of the desired propagation mode and the undesired attenuation mode. In this Figure 6b, with a 1 mm length open-ended waveguide, the simulation results showed that it significantly enhances the X-pol. isolation (or decoupling) from 4.9 dB to 17.5 dB.

**Figure 7:**
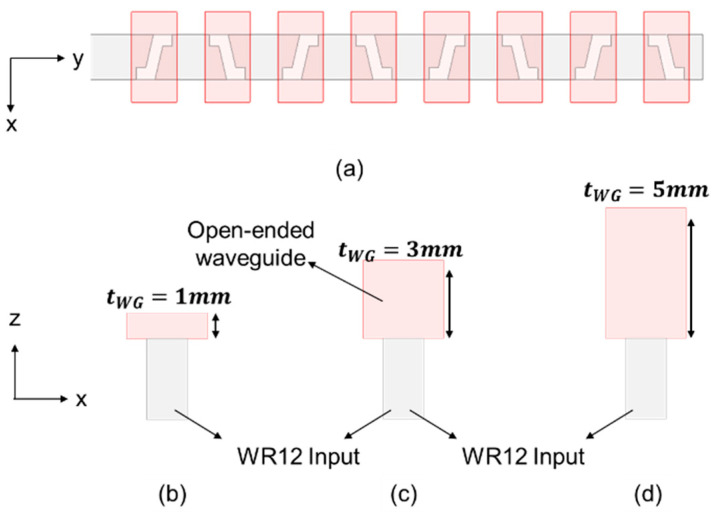
An *N* = 8 uniform slot arrays. (**a**) Top view; (**b**) front view, *t_WG_* = 1 mm; (**c**) front view, *t_WG_* = 3 mm; (**d**) front view, *t_WG_* = 5 mm.

In order to examine the relation between polarization purity and the length of the open-ended waveguide, uniform 8-slot arrays each with the same slot length and tilt angle but under different open-ended waveguide lengths were simulated; Figure 7a is the top view of this array, whilst Figure 7b–d demonstrate the front views of three different open-ended waveguide lengths (*t_WG_* = 1, 3, and 5 mm).

**Figure 8:**
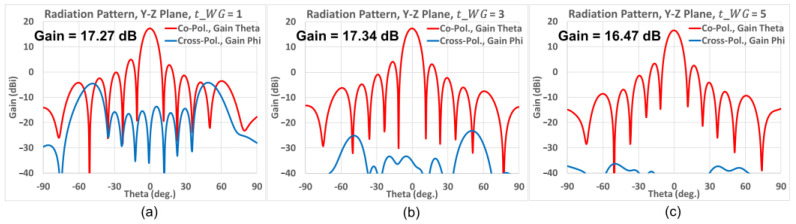
Simulated gain patterns in *y-z* plane at 77 GHz of a uniform 8-element array. (**a**) *t_WG_* = 1 mm; (**b**) *t_WG_* = 3 mm; (**c**) *t_WG_* = 5 mm. (Co-pol.: red line, X-pol.: blue line).

According to the simulated gain patterns of Figure 8, the red and blue lines, respectively, represent the co-pol. and X-pol. radiation patterns in the *y-z* plane at 77 GHz. The gain of the shortest case (*t_WG_* = 1 mm) was 17.23 dB but it exhibits poor X-pol. isolation at large scan angles, as Figure 8a shows. For the medium case (*t_WG_* = 3 mm), the gain is slightly increased to 17.32 dB and in addition, the X-pol. isolation at large scan angles is also improved dramatically, as seen from Figure 8b. As for the longest case (*t_WG_* = 5 mm), although it portrays the largest X-pol. isolation as shown in Figure 8c, its gain of 16.42 dB is the lowest among all three cases. Therefore, after making an assessment of these cases in terms of their gains and polarization purities, an open-ended waveguide with 3 mm length was chosen as the best tradeoff for the subsequent design studies, presented as follows.

Although the medium case (*t_WG_* = 3 mm) has a high gain and medium X-pol. isolation, the sidelobe level (SLL) between the main lobe and the first sidelobe is only 13 dB, which is not sufficient for many applications. Therefore, a concept based on the Chebyshev distribution is adopted to rectify this sidelobe issue, as will be discussed in the next section.

### 2.3. Chebyshev Distribution

The proposed structure here is a slotted waveguide antenna with 8 slots on the narrow wall. In order to reduce the sidelobe level for a uniform 8-element slot array, a method using the Chebyshev distribution is utilized, which possesses important properties that are able to suppress sidelobes and thus reach the condition of equal amplitude for every sidelobe. Theoretically, for an element in an array, the Chebyshev coefficient is larger when it is close to the center of the array; therefore, by controlling the coefficient, or so-called weighting, of each element properly, the proposed array can potentially attain equal amplitudes of all sidelobes [26,27].

A top view of the slot array is offered in Figure 7a. In order to simulate a realistic model, the open-ended waveguide is surrounded by metal. The Chebyshev coefficient corresponds to the power distribution of each element, and the power is proportional to the conductance from well-known formulas in basic circuit theory. Therefore, the relations of the Chebyshev coefficient between adjacent slots should be equal to the ratios of their conductance when the corresponding susceptance are zero. This condition of vanishing susceptance is called the resonance state. Under this condition, the antenna would match the LC resonance and be able to radiate properly. As mentioned in subsection A, there are two crucial variables that affect the characteristic relation of the slot, one of which is the slot length, and the other is the tilt angle (Figure 4b). For any given tilt angle *TA*, the relations of the conductance and susceptance with the slot length can be obtained, which can be repeated for other tilt angles.

**Figure 9:**
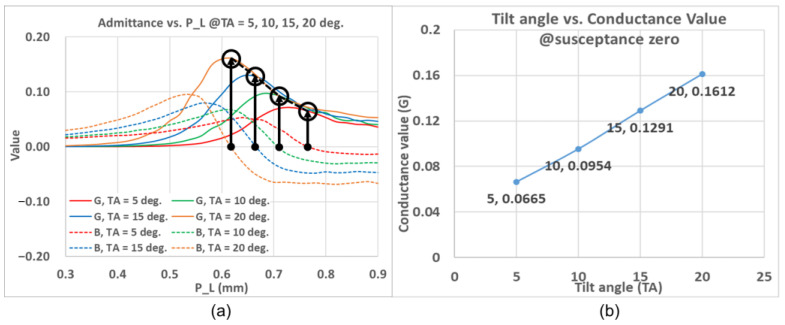
Characteristic relationship of slot parameters, (**a**) admittance (conductance and susceptance) with the tilt angle and the length of parallel part of the Z-shaped slot; (**b**) graph of conductance at zero susceptance versus the tilt angle.

Represented by solid and dotted traces, respectively, Figure 9a demonstrates an isolated slot with different *P_L* of the conductance and susceptance for four tilt angles ranging from 5° to 20° with a 5° step, each one pertaining to a certain trace color as indicated in the legend. For each *TA*, the condition of zero susceptance with associated *P_L* and conductance pertaining to the required Chebyshev coefficient is also indicated by the vertical black lines. As seen from the simulated results of Figure 9a, as the *TA* increases through values of 5°, 10°, 15°, and 20°, the corresponding conductance (G) of the slot is 0.0665, 0.0954, 0.1291, and 0.1612, respectively. With these combinations of *TA* and *P_L*, a look-up table pertaining to zero susceptance can be established, as given by Table 3. The matching of this system is normalized to the port impedance, which is the *TE*_10_ modal impedance of the waveguide, as the other modes are filtered out. For our antenna array comprising eight slots, the summation of the normalized conductance should be close to the value of one in order to reach resonance state. Besides, to simplify the variable numbers in the simulations, the *P_L* can be expressed as a function of the *TA* by interpolating the conductance of resonance state for four tilt angles in Figure 9a. To be specific, the variables of eight elements, i.e., four slot-pairs arrays, could reduce from eight (four *TA*, four *P_L*) to only four (*TA*). As another method to estimate slot parameters, the relation between the conductance (at zero susceptance) and the *TA* is established, as portrayed by Figure 9b. With a target conductance, one can estimate the *TA* by interpolation, upon which different *P_L* values can be swept through and searched for to meet the condition of zero susceptance.

**Figure 10:**
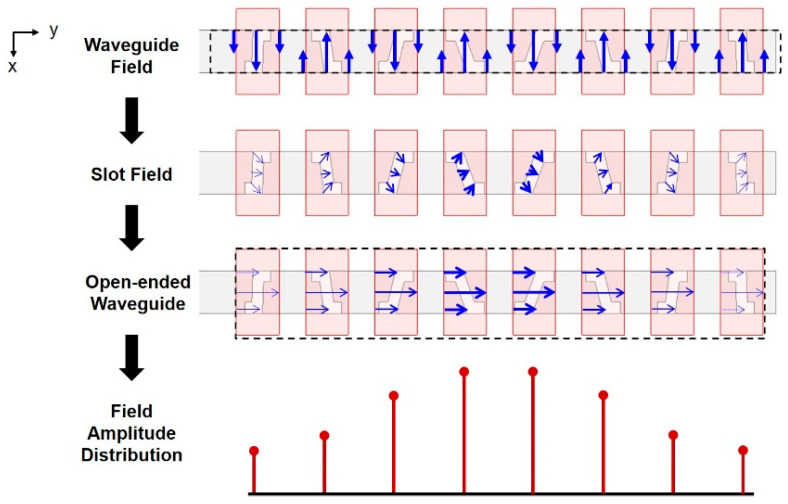
The propagation of electric field in the proposed narrow-wall slot waveguide antenna array. The blue arrow denotes the direction of electric field, and the width of an arrow denotes the amplitude of electric field.

To further describe the theory, Figure 10 demonstrates how the electric fields propagate through the structure.

As the horizontally *y*-directed *TE*_10_ modal *H*-fields on the side walls of the input waveguide generates *H*-fields with oppositely *y*-directed components within adjacent reversely-tilted Z-slots (separated by half the guide wavelength) but with co-oriented vertical *x* components, the corresponding inclined *E*-fields perpendicular to the aforementioned *H*-fields are induced inside the slots, such that the vertical *x* components in adjacent slots are oppositely oriented whilst the horizontal *y* ones are along each other. It is thus this latter co-oriented *E*-field component along with the abovementioned co-oriented *x*-directed *H*-field that is transported out by the exterior open-ended waveguide. After propagating through the open-ended waveguide, the *E* and *H* fields become horizontally *y* and vertically *x* directed, respectively. Combining the concept of Chebyshev distribution, the output amplitudes are shown at the bottom of Figure 10, which attain equal sidelobe levels.

## 3. Simulation Results

**Figure 11:**
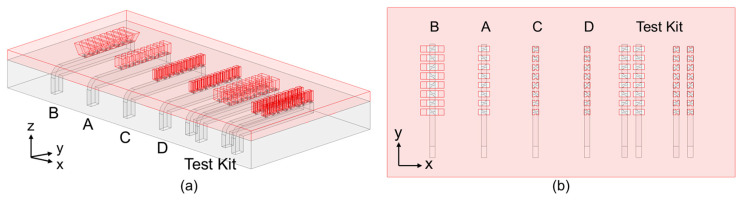
Four types of antenna array, (**a**) 3D view; (**b**) top view.

Four types of antenna array are simulated. The 3D view and top view of the simulation model are shown in Figure 11a and Figure 11b, respectively. More details of the procedure will be discussed in this section.

### 3.1. First Prototype (Type A)

**Figure 12:**
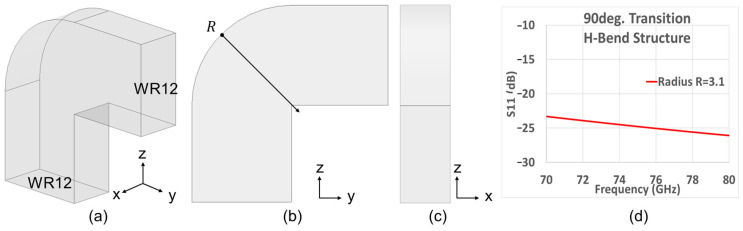
Simulation model and *S_11_* of the 90° transition H-bend structure. (**a**) 3D view; (**b**) side view; (**c**) front view; (**d**) simulated *S_11_* versus frequency.

Based on the theory in the preceding section, the antenna array was simulated. As subsequent experimentation of manufactured prototypes in our measurement chamber also has to be considered, the antenna array needs a 90° transition H-bend structure. Details of the measurement setup will be discussed in Section 4.2. The simulation model of the 90° transition structure is showed in Figure 12a–c. Parameter *R* = 3.1 mm represents the radius of the transition structure. The simulated variation with frequency of the reflection coefficient *S*_11_ for this structure over the considered band is given in Figure 12d, in which good matching is achieved.

**Figure 13:**
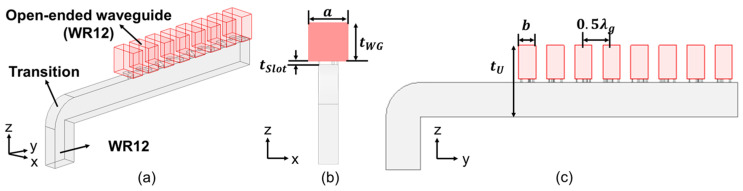
Simulation model of the first prototype Type A, (**a**) 3D view; (**b**) front view; and (**c**) side view.

For the first and simplest module, which we call Type A, we simulated an 8-element array with a WR12 as the open-ended waveguide, as schematized in Figure 13 with various views given by Figure 13a–c, the parameters of which are tabulated in Table 4.

**Figure 14:**
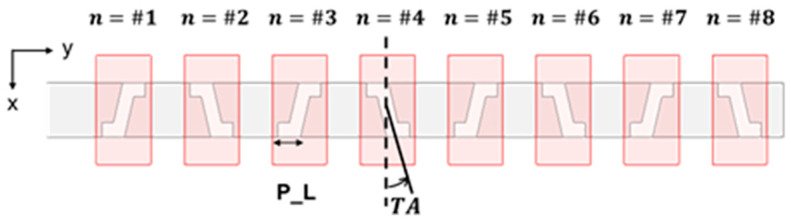
An 8-element array with Chebyshev distribution, Type A prototype.

For an 8-element array, to obtain a maximal relative SLL of −20 dB, the Chebyshev coefficients, expressed as magnitudes of power, are listed in Table 5. The ratios of the Chebyshev coefficient between each adjacent pair of slots are the same as the ratio of their conductance. After fine-tuning the slot angles based on 8-element Chebyshev −20 dB SLL condition, the slot array is depicted in Figure 14, and the parameters of each slot are listed in Table 6. As can be seen, moving from the center element towards either side of the array, the *TA* and *P_L*, respectively, decreases and increases in accordance with the falling Chebyshev coefficient and thus the conductance as well. This conforms with Figure 9, in which the moving down from higher to lower conductance values is associated with the reduction in the *TA* as well as the increment in the parallel arm length of the Z-slot.

**Figure 15:**
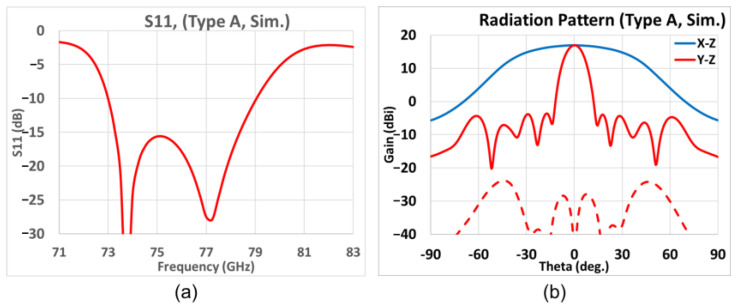
Simulation results of Type A prototype with Chebyshev distribution. (**a**) *S*_11_ over frequency band; (**b**) far- field radiation pattern at 77 GHz (red and blue solid traces represent co-polar gains in elevation *y-z* and azimuth *x-z* planes, respectively; red-dashed trace gives X-pol. radiation in elevation *y-z* plane).

As the simulated result of Figure 15a shows, the −10 dB matching bandwidth at 77 GHz is 7.8% in the frequency band. The simulated gain patterns at 77 GHz in the elevation (*y-z*, red line) and azimuth (*x-z*, blue line) planes are presented in Figure 15b; in addition, the solid line represents the co-pol. and the dash line represents the X-pol. The elevation plane contains the periodic alignment of the array and thus portrays the strongly directive beam pattern. The maximum gain towards the broadside (*θ* = 0°) is 17.0 dBi, with SLL lower than that by 20.6 dB in the narrower beam pattern of the *y-z* plane. The 3 dB BW in the elevation (*y-z*) and azimuth (*x-z*) planes are 11.4°and 68.7°, respectively, and the 6 dB azimuth BW is 88.9°. The X-pol. isolation is over 20 dB in both the elevation and azimuth planes, and the X-pol. in the azimuth plane is below −40 dB.

In the previous section, the SLL of a uniform 8-element array without the use of the Chebyshev distribution is about 15 dB (Figure 8b). Therefore, the benefit of Chebyshev distribution is that it offers a 5 dB reduction of the sidelobe level.

**Figure 16:**
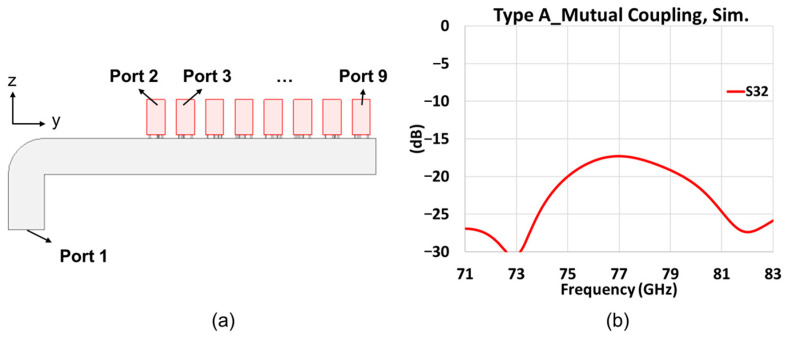
Mutual coupling between elements within an array, (**a**) simulation model; (**b**) simulated *S*_32_.

In order to check the mutual coupling between elements within an array, a model with ports at the end of each open-ended waveguide is simulated. The model and result are shown in Figure 16a and Figure 16b, respectively. The mutual coupling between the first and second elements is below −17 dB at 77 GHz. As the separation distance becomes larger, the third to ninth element has an even smaller mutual coupling effect on the first element.

**Figure 17:**
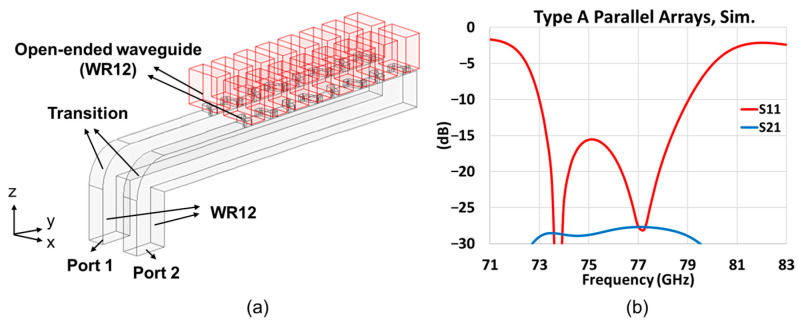
Test kit of two Type A arrays with an interval of a free space wavelength at 77 GHz, (**a**) simulation model; (**b**) simulation results (*S*_11_: red line; mutual coupling: blue line).

The mutual coupling between adjacent arrays is also investigated. Towards this, two test kits are designed on the right side of the model as shown in Figure 11b. One of the test kits examines two Type A arrays with an interval of a free space wavelength *λ*_0_ of 3.9 mm at 77 GHz, as given in Figure 17a. The simulation results in Figure 17b show that the insertion loss *S*_21_ (blue line) is lower than −25 dB, which means the effect of the adjacent array is minor. Hence, the *S*_11_ (red line) is highly similar to a single Type A array, as can be concluded upon comparison with Figure 15a.

**Figure 18:**
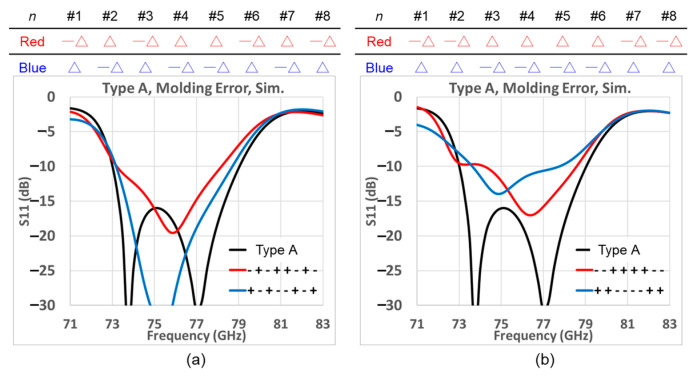
Sensitivity of matching to manufacture tolerances, with distributions of errors among the eight array elements as tabulated, each perturbation quantified by Δ = 0.05 mm. (**a**) Odd and even pair of slots with different signs of molding error; (**b**) two of inner and outer pairs of slots with different signs of molding error (red and blue solid traces represent the molding error arrangements with opposite signs).

The sensitivity of the design performance to manufacturing inaccuracies could also be of interest. The molding tolerance error for the metallic waveguide is about 0.05 mm in general. Therefore, we have simulated the return losses with two different molding error distributions. For the Type A design of Figure 14, each of the following two tables in Figure 18 shows an arbitrary distribution of errors introduced to the *P_L* of the array elements, the error of any one element quantified by the positive or negative of a symbol Δ which stands for a molding error of 0.05 mm. The graph below each tabulated error distribution, i.e., Figure 18a,b, portrays the corresponding variations of the reflection with frequency, in which the black trace pertains to the idealized case, whereas the red and blue lines are the results with molding errors according to the distribution of the ±Δ listed in the corresponding table. As there are two pairs (red & blue) of error distributions per table per graph, and there are two tables and their two graphs, a total of four distinct error distributions are shown. According to the results, although the quality of matching expectedly changes with molding errors in the order of ±0.05 mm, the −10 dB bandwidth still covers the designated bandwidth of 76–77 GHz.

### 3.2. Different Antenna Types for Diverse Applications

**Figure 19:**
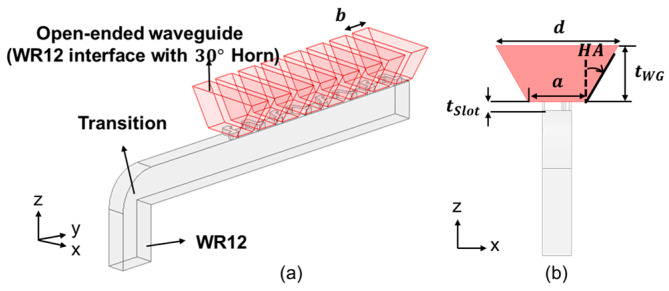
Simulation model of Type B prototype with open-ended H-plane sectoral pyramidal horns with flare angle 30°, (**a**) 3D view; (**b**) front view.

Building upon the study of the first prototype, which we have called Type A, of the preceding subsection, we proceed to the next design concept, which we call Type B, again with 8 radiating slot elements, but now with the former WR12 rectangular open-ended waveguides replaced by open-ended H-plane sectoral pyramidal horns with a 30° flare angle, as portrayed by Figure 19, and whose parameters are tabulated in Table 7.

**Figure 20:**
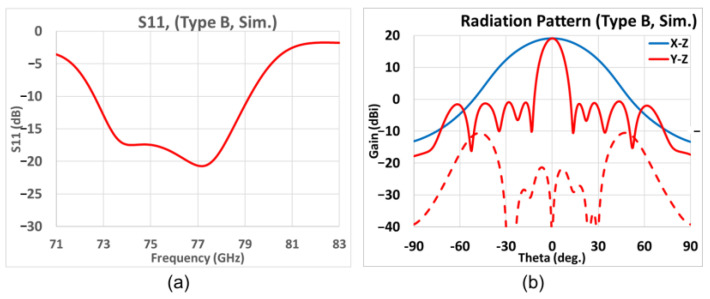
Simulation results of Type B. (**a**) *S*_11_ over frequency band; (**b**) far-field radiation pattern at 77 GHz (red and blue solid traces represent co-polar gains in elevation *y-z* and azimuth *x-z* planes, respectively; red-dashed trace gives X-pol radiation in elevation *y-z* plane).

For arrays with larger aperture sizes, the gain would be higher and the beamwidth would be narrower. The *TA* and *P_L* are the two parameters that can be tailored towards optimal performances in terms of the matching bandwidth, gain, sidelobes, and beamwidths in the elevation and azimuth planes, several parametric values of which, each pair indexed by *n*, are tabulated in Table 7. The fine-tuned slot case is that of *n* = 8 in Table 8, for which the −10 dB bandwidth at 77 GHz is wider than 8%, as Figure 20a shows, and with an associated maximum broadside gain of 19.0 dBi with SLL relative to that of the main beam being −19.7 dB at 77 GHz, as observed from the gain patterns in the elevation (*y-z*, red line) and azimuth (*x-z*, blue line) planes of Figure 20b. Solid line and dash line also stands for co-pol. and X-pol. The 3 dB beamwidth in the elevation and azimuth planes are, respectively, 11.0° and 40.1°, and the 6 dB beamwidth in the azimuth plane is 56.8°. The X-pol. isolation in the elevation plane is over 20 dB within ±40°, and the cross-polar gain in the azimuth plane is below −40 dB.

**Figure 21:**
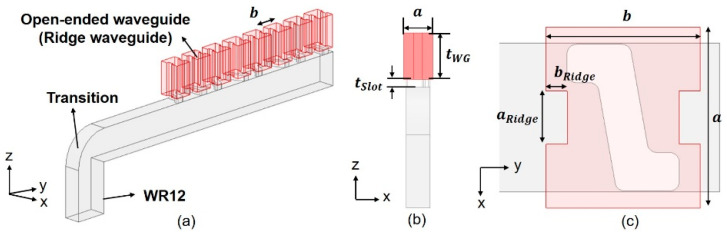
Simulation model of Type C prototype with smaller radiating apertures, (**a**) 3D view; (**b**) front view; (**c**) top view of slot.

Converse to the increment of the aperture size of the open-ended waveguides for enhanced gain and narrowed beamwidth, decreasing the aperture leads to a reduced directivity and widened beamwidth. Towards this end, the upcoming third design, called Type C, entails an open-ended ridged waveguide with a smaller radiating aperture that is placed over each slot. The perspective view is given in Figure 21a,b, while the unit cell of one slot portraying the ridged waveguide is shown in Figure 21c. The dimension of the ridged waveguide is *a* × *b* = 1.88 × 1.60 (mm^2^). The parameters are given in Table 9.

**Figure 22:**
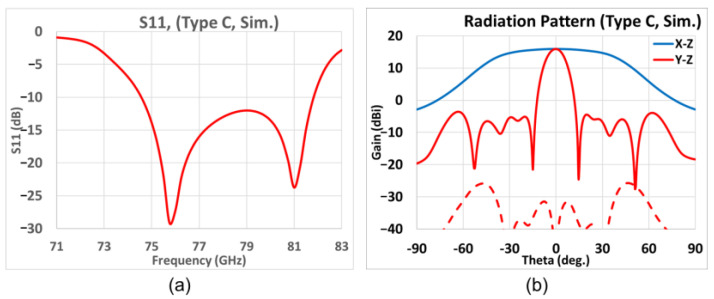
Simulation results of Type C. (**a**) *S*_11_ vs. frequency; (**b**) far-field radiation pattern at 77 GHz (red and blue solid traces represent co-polar gains in elevation *y-z* and azimuth *x-z* planes, respectively; red-dashed trace gives X-pol radiation in elevation *y-z* plane).

The fine-tuning *TA*, *P_L*, and ridge dimensions are given in Table 10, of which the simulated graph of *S*_11_ versus frequency, and the far-field gain patterns in the two principal *(y-z* and *x-z*) planes at 77 GHz are presented in Figure 22a, and Figure 22b, respectively. Figure 22a shows sufficient matching and shows that the −10 dB bandwidth at 77 GHz is over 9%. As seen from the gain pattern in the elevation (*y-z*, red line) and azimuth (*x-z*, blue line) planes of Figure 22b, the maximum broadside gain is 16.0 dB with sidelobe levels being 19.5 dB below the main beam. The 3 dB BWs in the elevation and azimuth planes are, respectively, 11.9°and 80.1°, while the 6 dB BW in the azimuth plane is 99.1°, being broader beamwidths than the previous cases as expected. The X-pol. isolation in both elevation and azimuth planes show good isolation levels over −20 dB.

For the final application, referred to as Type D, the open-ended waveguides are ridged as well. The only difference here is that the side length a is almost ten percent shorter compared to that of Type C. In other words, the dimensions of the ridged waveguide of Type D are 1.70 × 1.60 (mm^2^), and the parameters are given in Table 11.

**Figure 23:**
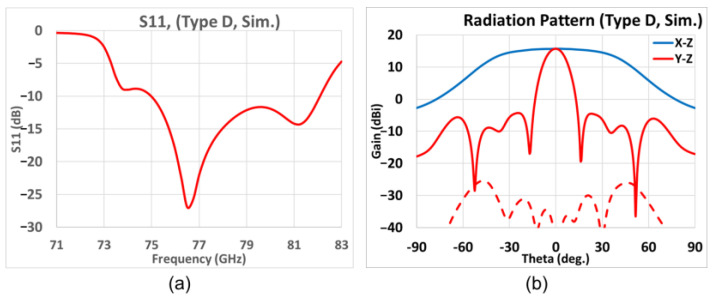
Simulation results of Type D. (**a**) Frequency variation of *S*_11_; (**b**) far-field radiation pattern at 77 GHz (red and blue solid traces represent co-polar gains in elevation *y-z* and azimuth *x-z* planes, respectively; red-dashed trace gives X-pol radiation in elevation *y-z* plane).

A fine-tuning slot length *P_L* and the tilt angle *TA* for the span of parametric values given in Table 12, the simulated variation of the *S_11_* with frequency of Figure 23a shows the good matching in the frequency band, and the −10 dB bandwidth centered at 77 GHz is about 9%. As the simulated gain patterns at 77 GHz in the elevation (*y-z*, red line) and azimuth (*x-z*, blue line) planes of Figure 23b show, the maximum gain is 15.7 dB with sidelobe levels being 20.0 dB below the main beam level. The 3 dB beamwidth in the elevation (*y-z*) plane is 12.8°, whereas that in the azimuth (*x-z*) plane is 81.2°. The 6 dB beamwidth in the azimuth plane is 100.2°. The X-pol. isolation in both elevation and azimuth planes show good isolation levels over −20 dB.

### 3.3. Summary of the Proposed Antennas

To sum up the simulation results of the proposed four types of antenna array designed at a center frequency of 77 GHz, a summarized table is given in Table 13. Aspects such as the aperture size, −10 dB fractional bandwidth (FBW), peak gain, SLL, 3 dB elevation beamwidth (3 dB EL BW), 3 dB azimuth beamwidth (3 dB AZ BW), and 6 dB azimuth beamwidth (3 dB AZ BW) are listed.

## 4. Measurement

### 4.1. CNC Sample

**Figure 24:**
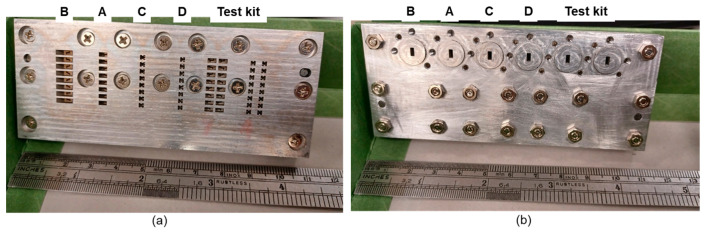
Photographs of manufactured CNC sample test board. (**a**) Top view; and (**b**) bottom view.

Based on the designs established by the foregoing simulation results, a CNC sample was manufactured by aluminum material. The top and bottom views of the prototype are photographed in Figure 24a and Figure 24b, respectively. We measured one type at a time, and a WR12 input is connected to the bottom side which excites the radiation field from the top layer.

### 4.2. Measurement Setup

**Figure 25:**
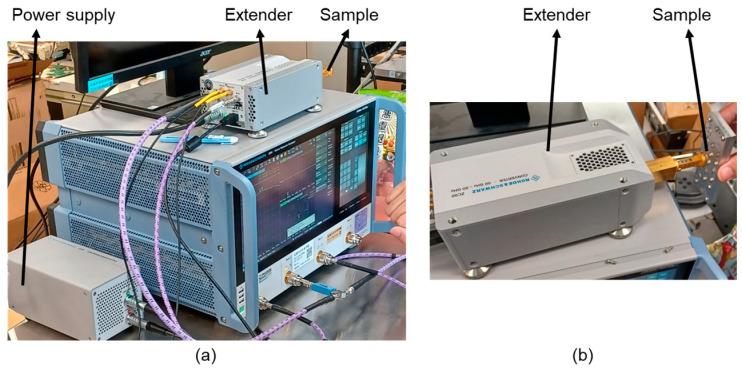
Photographs of measurement setup of *S*_11_. (**a**) The overall setup; and (**b**) connection of sample and extender.

The measurement environment of *S*_11_ was shown in Figure 25. A power supply, vector network analyzer, and extender are needed, and the setup was one port calibration with WR12 calibration kits (Rohde & Schwarz GmbH & Co. KG, Munich, Bavaria, Germany).

**Figure 26:**
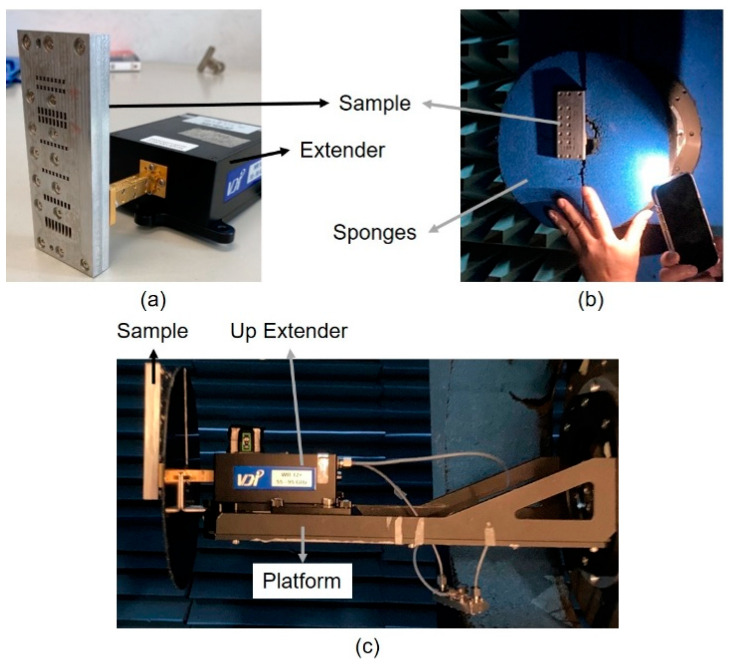
Photographs of setup for measurement of radiation pattern. (**a**) Connection of sample and down extender; (**b**) front view in CATR; (**c**) side view in CATR chamber.

The measurement environment of the radiation pattern was set up in a Compact Antenna Test Range (CATR) chamber room (WavePro, Inc., Taoyuan City, Taiwan). The input feeding structure was excited by an up extender and a down extender (Virginia Diodes, Inc., Charlottesville, VA, USA), as shown in Figure 26a. Moreover, sponges were placed between the sample and extender to avoid the interference by metal objects like fixture, extender or platform, as shown in Figure 26b. The antenna sample was placed on the platform in the CATR chamber, as shown in Figure 26c.

### 4.3. Measurement Results

**Figure 27:**
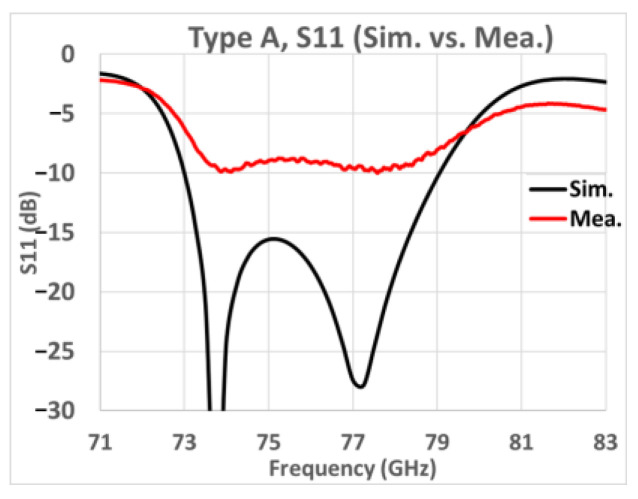
Type A, simulated and measured *S*_11_ (black line: simulation; red line: measurement).

Although our sample included four types of arrays, we took Type A as a representative. In Figure 27, the shape of the *S*_11_ variation with frequency shows that the simulation and measurement results have similar frequency responses although the average level of the measured reflection coefficient is −9 dB from 76 to 77 GHz.

**Figure 28:**
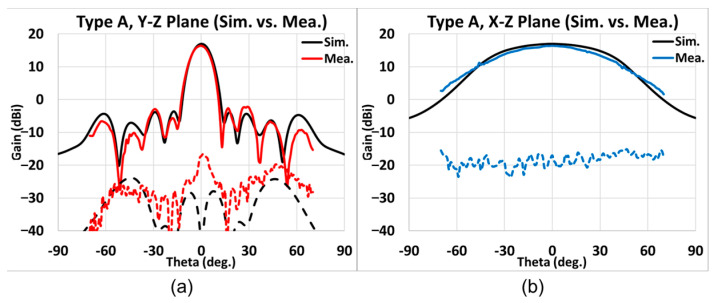
Type A, simulated and measured far-field radiation patterns at 77 GHz. (**a**) Elevation plane; (**b**) azimuth plane (black line: simulation results; red/blue line: measurement results; solid line: co-pol.; dash line: X-pol.).

**Figure 29:**
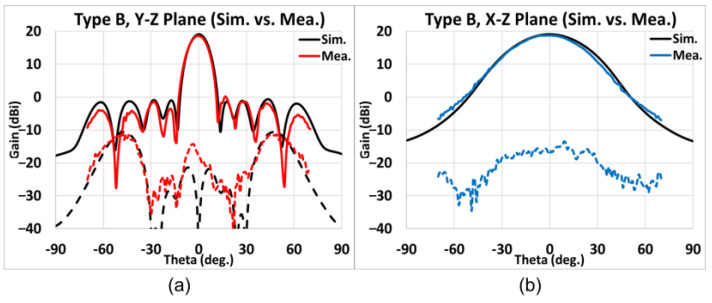
Type B, simulated and measured far-field radiation patterns at 77 GHz. (**a**) Elevation plane; (**b**) azimuth plane (black line: simulation; red/blue line: measurement; solid line: co-pol.; dash line: X-pol.).

**Figure 30:**
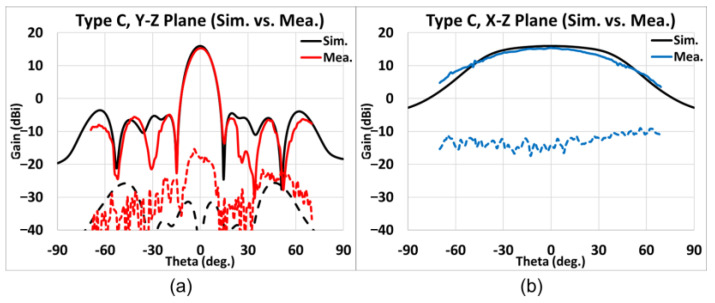
Type C, simulated and measured far-field radiation patterns at 77 GHz. (**a**) Elevation plane; (**b**) azimuth plane (black line: simulation results; red/blue line: measurement results; solid line: co-pol.; dash line: X-pol.).

**Figure 31:**
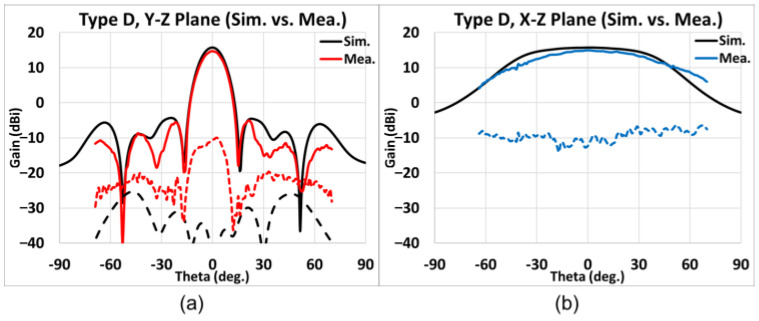
Type D, simulated and measured far-field radiation patterns at 77 GHz. (**a**) Elevation plane; (**b**) azimuth plane (black line: simulation; red/blue line: measurement; solid line: co-pol.; dash line: X-pol.).

Presented in Figure 28, Figure 29, Figure 30 and Figure 31 is the simulated (black line) and measured (elevation plane: red line; azimuth plane: blue line) radiation patterns at 77 GHz of Types A, B, C, and D, respectively. In addition, the solid line represents the co-pol., and the dash line represents the X-pol.

For Type A, the elevation and the azimuth plane results are shown in Figure 28a,b. The maximum simulated gain is 16.97 dB while the measured average maximum gains of the H-cut and V-cut is 16.41 dB. Thus, the gain difference with simulation is −0.56 dB. The SLL is −18.5 dB, and the X-pol. isolation is lower than −20 dB; additionally, the 3 dB beamwidth in the elevation (*y-z*) plane is 11°, whereas that in the azimuth (*x-z*) plane is 62°, and the 6 dB beamwidth in the azimuth plane is 87°.

For Type B, the elevation and the azimuth plane results are shown in Figure 29a,b. The maximum simulated gain is 19.05 dB while the measured average maximum gains of the H-cut and V-cut is 18.71 dB. Thus, the gain difference is −0.34 dB and the SLL is −18.5 dB. The X-pol. isolation is greater than 20 dB within ±40°. In addition, the 3 dB beamwidth in the elevation (*y-z*) plane is 11°, whereas that in the azimuth (*x-z*) plane is 38°. The 6 dB beamwidth in the azimuth plane is 54°.

For Type C, the elevation and the azimuth plane results are shown in Figure 30a,b. The maximum gain of simulations is 15.97 dB while the measured average maximum gains of the H-cut and V-cut is 15.37 dB. Therefore, the gain difference is −0.60 dB, and the SLL is −20.0 dB. The X-pol. isolation is stronger than 20 dB. The 3 dB beamwidth in the elevation (*y-z*) plane is 12°, whereas that in the azimuth (*x-z*) plane is 73°. The 6 dB beamwidth in the azimuth plane is 103°.

For Type D, the elevation and the azimuth plane results are shown in Figure 31a,b. The maximum simulated gain is 15.69 dB while the measured average maximum gains of the H-cut and V-cut is 14.87 dB; so, the gain difference is about −0.82 dB. The SLL is −19.5 dB and the X-pol. isolation is acceptable within the main lobe. The 3 dB beamwidth in the elevation (*y-z*) plane is 13°, whereas that in the azimuth (*x-z*) plane is 72°. The 6 dB beamwidth in the azimuth plane is 107°.

### 4.4. Comparison of Performance with Existing Literatures

Finally, some previous works on antenna arrays of similar calibers operating within the same W-band of the millimeter wave regime as studied here are herein compared, as shown in Table 14. We chose Type A with an open-ended waveguide to represent this work as a benchmark; in addition, eight aspects are considered, such as antenna type, element number, aperture dimension, FBW with respect to the center frequency, gain, SLL, 3 dB EL BW, and radiation efficiency.

Because this work proposed a 1 by 8 linear array, the configurations of the other articles in the literature on likewise linear arrays that have been selected for comparison are either 1 by 8 or 1 by 10. Among these works, the FBW of our design is wider than those in [29,30,33] (not clear in [31]), and the gain outdoes the others no matter in simulations or measurements. However, the proposed array was set under the scenario of eight elements that are subjected to the Chebyshev condition administered for a −20 dB SLL criterion only (and not something lower), so the SLL suppression was understandably lower than other works’ simulation results, but the sidelobe value is still acceptable. Had a more stringent (lower) SLL criterion for the Chebyshev coefficients been prescribed, our sidelobe suppression will not be inferior to those of the others. As for the aspect of 3 dB EL BW, either of our simulated or measured performance surpasses those of [28,30,31,32,33]. As for the radiation efficiency, the measurement result of this work is calculated to be 87%, which is 1% less than the simulation result of [32] and higher than the measurement result in [33] by 12%. Although the dimension of the proposed array seems larger, the narrow-wall array provides a compact size that affords a tighter arrangement for the placements of adjacent arrays.

## 5. Conclusions

A multi-application 1 × 8 narrow-wall slotted waveguide antenna array is presented, which can be applied to 5G and 6G technologies, as well as automobile systems. This antenna array has low propagation loss due to the usage of a waveguide as a propagation medium. Embedded Z-shaped slots on the narrow-wall and inclined with alternating opposite tilt angles offer enhanced gain while affording shortened unit cells that fulfill the requirement of keeping within $0.5\lambda_{g}$ element spacing. The side-by-side test-kit demonstrates low coupling between adjacent 1 × 8 arrays. These characteristics offer the potential of making compact antenna arrays. Moreover, the open-ended waveguide placed over each slot element provides high polarization performance as the electric field propagates through the slot layer. The Chebyshev distribution is also implemented to suppress sidelobe radiation. With the aforementioned features and the comparison with the existing literature, a high gain antenna array is achieved. Last but not least, the simulated and measured radiation patterns demonstrate mutual consistency and agreement. The peak gain difference is only 0.56 dB and the sidelobe level remains at levels ranging from −18.5 dB to −19.0 dB.

## Figures and Tables

**Table 1:** Loss difference between rectangular waveguide and microstrip line [8].

Prototype (Frequency)	Simulated Loss (dB/cm)	Measured Min-Max Loss (dB/cm)
Rectangular waveguide (50–75 GHz)	0.0136	0.0295–0.0420
Microstrip (50–75 GHz) 0.127–0.200 mm substrate	Rogers 4003: 0.271	0.7055

**Table 2:** The comparison of different slot shapes.

Ref.	Slot	Narrow/ Broad Wall	Slot Spacing	$S_{11}$, −10 dB BW	Advantages/ Disadvantages
[19] (2013)	Spiral	Broad wall	$\lambda_{g}$	35%	Wideband Stable gain Elliptical polarization Hard to fabricate
[20] (2019)	V-shaped	Broad wall	$0.5\lambda_{g}$	5.5%	Pure polarization Compact Narrow bandwidth Hard to fabricate
[21] (2019)	T-shaped	Broad wall	$0.5\lambda_{g}$	3.4%	High gain Circular polarization Compact Narrow bandwidth Hard to fabricate
[22] (2021)	Split-ring	Broad wall	$\lambda_{g}$	30.5%	Wideband High gain Beam steering Hard to fabricate
[14] (2015)	I-shaped	Narrow wall	$0.5\lambda_{g}$	5%	Compact slot without tilting Hard to fabricate dipoles Lossy structure
This work	Z-shaped	Narrow wall	$0.5\lambda_{g}$	7.8%	Compact Easy design High gain

**Table 3:** Look-up table pertaining to zero susceptance for several combinations of *TA* and *P_L*.

*TA* (deg)	*P_L* (mm)	Conductance (G)	Susceptance (B)
5	0.76	0.0665	0
10	070	0.0954	0
15	0.66	0.1291	0
20	0.62	0.1612	0

**Table 4:** Parameter of Type A prototype (unit in mm).

$\lambda_{g}$	a	b	$t_{WG}$	$t_{Slot}$
5.01	~3.10	~1.55	3	0.5

**Table 5:** The Chebyshev coefficient of 8 elements under −20 dB sidelobe level condition.

*n*	#1	#2	#3	#4	#5	#6	#7	#8
Coefficient in Power	1.00	1.30	2.28	2.97	2.97	2.28	1.30	1.00

**Table 6:** Parameters of Chebyshev-distributed 8-element array of Figure 14 Type A prototype.

*n*	#1	#2	#3	#4	#5	#6	#7	#8
*TA* (°)	1.1	3.3	16.7	20.8	20.8	16.7	3.3	1.1
*P_L* (mm)	0.8	0.8	0.6	0.6	0.6	0.6	0.8	0.8

**Table 7:** Parameters of waveguide portion of Type B prototype (unit in mm).

$\lambda_{g}$	a	b	d	$t_{WG}$	$t_{Slot}$
5.01	~3.10	1.80	~6.56	3	0.5

**Table 8:** Parameters of slot of Type B.

*n*	#1	#2	#3	#4	#5	#6	#7	#8
*TA* (°)	1.3	1.4	17.3	20.5	20.5	17.3	1.4	1.3
*P_L* (mm)	0.8	0.8	0.6	0.6	0.6	0.6	0.8	0.8

**Table 9:** Parameters of waveguide portion of Type C prototype (unit in mm).

$\lambda_{g}$	a	b	$t_{WG}$	$t_{Slot}$	$a_{Ridge}$	$b_{Ridge}$
5.01	~1.88	1.6	3	0.5	0.55	0.22

**Table 10:** Parameters of slot of Type C prototype with smaller radiating apertures.

*n*	#1	#2	#3	#4	#5	#6	#7	#8
*TA* (°)	2.9	2.0	24.9	24.8	24.8	24.9	2.0	2.9
*P_L* (mm)	0.7	0.8	0.6	0.6	0.6	0.6	0.8	0.7

**Table 11:** Parameters of waveguide portion of Type D prototype. Dimensions are the same as those in Figure 21c (unit in mm).

$\lambda_{g}$	a	b	$t_{WG}$	$t_{Slot}$	$a_{Ridge}$	$b_{Ridge}$
5.01	1.7	1.6	3	0.5	0.40	0.30

**Table 12:** The parameters of slot portion of Type D.

*n*	#1	#2	#3	#4	#5	#6	#7	#8
*TA* (°)	2.9	2.0	24.9	24.8	24.8	24.9	2.0	2.9
*P_L* (mm)	0.7	0.8	0.6	0.6	0.6	0.6	0.8	0.7

**Table 13:** Summary table of proposed antennas.

Type	A	B	C	D
Aperture size (${mm}^{2}$)	4.8	11.8	3.0	2.7
−10 dB FBW (%)	7.9	8.6	9.4	9.3
Peak gain (dB)	17.0	19.0	16.0	15.7
SLL (dB)	−20.6	−19.7	−19.5	−20.0
3-dB EL BW (°)	11.4	11.1	11.9	12.8
3-dBAZ BW (°)	68.7	40.1	80.1	81.2
6-dBAZ BW (°)	88.9	56.8	99.1	100.2

**Table 14:** Comparison with existing literature.

REF.	Antenna Type	Element Number	Aperture Dimension (*λ*_0_^2^)	FBW (%) @ Central Frequency (GHz)	Gain (dBi)	SLL (dB)	3 dB EL BW ( °)	Radiation Efficiency
[28] (2023)	Patch	1×10	0.49×0.26	<6.5%, 77 GHz (Sim.)	13.8 (Sim.)	−24.4 (Sim.)	10.0 (Sim.)	N.A.
[29] (2023)	Patch	1×8	0.35×0.27	4.1%, 79 GHz (Sim.) 1.7%, 79 GHz (Mea.)	15.1 (Mea.)	N.A.	11.7 (Mea.)	N.A.
[30] (2011)	Patch	1×10	0.29×0.25	1.9%, 77 GHz (Sim.)	16 (Sim.)	−22 (Sim.)	10 (Sim.)	N.A.
[31] (2018)	Slot SIW	1×8	N.A.	>4%, 76.5 GHz (Sim.)	14.4 (Sim.)	−19.2 (Sim.)	10.2 (Sim.)	N.A.
[32] (2024)	Patch	1×8	0.43×0.26	44.2%, 64 GHz (Sim.) 20.3%, 64 GHz (Mea.)	13.5 (Sim.)	−10 (Sim.)	6.4 (Sim.)	88% (Sim.)
[33] (2024)	Slot RGW	1×8	0.44×0.44	2.2%, 77 GHz (Sim.) 2.2%, 77 GHz (Mea.)	16 (Sim.) 15 (Mea.)	−10 (Sim.) −10 (Mea.)	10 (Sim.) 10 (Mea.)	75% (Mea.)
This Work	Slot Waveguide	1×8	0.80×0.40	7.8%, 77 GHz (Sim.)	17.0 (Sim.) 16.4 (Mea.)	−20.6 (Sim.) −18.5 (Mea.)	11.4 (Sim.) 11 (Mea.)	87% (Mea.)

## Data Availability

The data supporting this study are included within the article.
